# Thoracobiliary fistulas: literature review and a case report of fistula closure with omentum majus

**DOI:** 10.2478/raon-2013-0003

**Published:** 2013-02-01

**Authors:** Anton Crnjac, Vid Pivec, Arpad Ivanecz

**Affiliations:** 1 Department of Thoracic Surgery, University Clinical Center Maribor, Maribor, Slovenia; 2 Department of Abdominal Surgery, University Clinical Center Maribor, Maribor, Slovenia

**Keywords:** thoracobiliary fistula, bronchobiliary fistula, treatment, omentum majus

## Abstract

**Background:**

Thoracobiliary fistulas are pathological communications between the biliary tract and the bronchial tree (bronchobiliary fistulas) or the biliary tract and the pleural space (pleurobiliary fistulas).

**Review of the literature:**

We have reviewed aetiology, pathogenesis, predilection formation points, the clinical picture, diagnostic possibilities, and therapeutic options for thoracobiliary fistulas.

**Case report:**

A patient with an iatrogenic bronchobiliary fistula which developed after radiofrequency ablation of a colorectal carcinoma metastasis of the liver is present. We also describe the closure of the bronchobiliary fistula with the greater omentum as a possible manner of fistula closure, which was not reported previously according to the knowledge of the authors.

**Conclusions:**

Newer papers report of successful non-surgical therapy, although the bulk of the literature advocates surgical therapy. Fistula closure with the greater omentum is a possible method of the thoracobiliary fistula treatment.

## Introduction

In the case of bronchobiliary (BBF) and pleurobiliary fistulas (PBF), there is an existing pathological communication between the biliary tract and bronchial tree in the first case and pleural space in the second.

The literature indicates several possible *causes* for the conditions, but all potential causes can be summarized in five groups:^1^–^5^

1. Congenital bronchobiliary or pleurobiliary fistulas;
  fistulas that are the result of
2. hepatic hydatid disease or liver abscess (echinococcic, amoebic, pyogenic),
3. biliary tract obstruction (iatrogenic cause or trauma excluded),
4. injury (blunt or penetrant) and
5. iatrogenic fistulas (liver resection, radiofrequency ablation - RFA, bile duct stricture, irradiation, thoracic drainage, etc…).

Pathogenesis may be, except in the case of the congenital form of the disease, explained by two mechanisms.^2^,^4^,^6^ In the first case biliary tract obstruction is the primary reason for fistula formation. Causes may be scars (trauma, surgery, after radiation, etc…), inflammatory diseases, foreign bodies, primary tumours, metastases or granulomas of different aetiologies, which obstruct the bile ducts. The result is the retention of bile proximal to the barrier, the formation of a liver biloma and subsequently the abscess formation. By increasing, the abscess gradually erodes the diaphragm. In case of adhesions between the lower lung lobe and the diaphragm (due to previous pleural or lung pathology), the abscess erodes directly into the lung parenchyma until it reaches the nearest bronchus and a BBF is formed. When no previous pleural pathology is present, the abscess gradually erodes into the pleural space and a PBF with pleural empyema is formed.

In the second case, the formation of a thoracobiliary fistula (TBF) takes place without biliary tract obstruction. In this case a hydatid *cyst* or a *liver abscess* is the primary reason for the fistula formation. The abscess can be echinococcic (most often), amoebic, or pyogenic in origin. As described above, the cyst or the abscess gradually enlarge and erode the diaphragm. Depending on the previous state of the pleural space, a BBF or a PBF are formed. The *predilection point* for the fistula formation is the posteromedial part of the right hemidiaphragm- *i.e*. the part which is in a direct contact with the area nuda hepatis.^7^

Because BBF and PBF are rare phenomena, larger studies on their frequency and the most common causes do not exist. In the literature, liver abscess of echinococcic origin or hydatid disease of the liver are stated as the most frequent causes in the developing countries and also globally.^2^,^3^,^5^,^6^,^8^,^9^ Information about the most common cause in the developed world is contradictory (Table 1).^1^,^2^,^4^,^5^,^7^,^10^–^25^

The clinical picture of patients with BBF and PBF may be present either in the acute or chronic form.^2^,^7^ Symptoms result from the underlying disease of the liver, biliary tract, and lung pathology. Bilioptysis, whenever encountered, is pathognomonic for bronchobiliary fistula.^2^,^3^,^7^,^8^,^10^,^21^ In the case of the acute form^2^,^7^, the patient is in distress, with elevated body temperature, and complains of pain in the lower right part of the chest. In the case of BBF, bilioptysis is present and during physical examination, inspiratory crackles over the lower-right parts of the lung are noticed. The fulminant disease presents in the form of acute respiratory distress syndrome (ARDS). When the patient has a PBF, the cough is dry and irritating, with the absence of respiratory phenomena above the right bottom of the lungs. A chronic TBF^2^,^7^ presents with dry, irritating cough, occasional yellowish sputum, intermittent fever with malaise, and weight loss. The clinical picture mimics recurrent pneumonia.

Beside the pulmonary symptoms there are also symptoms due to the underlying disease of the liver and biliary system.^2^,^7^ There might be upper right quadrant abdominal pain that irradiates to the right shoulder. Jaundice in the case of bile duct obstruction. Sometimes a biliocutaneus fistula can also be encountered.

When examining the laboratory results elevated white cell blood count and C-reactive protein (CRP) is noticed. Anaemia and hypoalbuminaemia are signs of chronic TBF. In the case of bile duct obstruction, elevated levels of direct and total bilirubin are present.^2^ The *microbiological analysis* of sputum reveals the presence of the following microbes: E. coli, Klebsiella spp., Pseudomonas aerug., Enterococcus spp. and Enterobacter cloacae.^3^,^8^,^17^

On chest x-ray a raised right hemidiaphragm, shadowing in the area of the lower and middle right lobus, atelectasis and pleural effusion might be seen.^6^,^9^ Occasionally, gas-fluid levels are present on abdominal x-ray images.^4^,^8^

**FIGURE 1.**
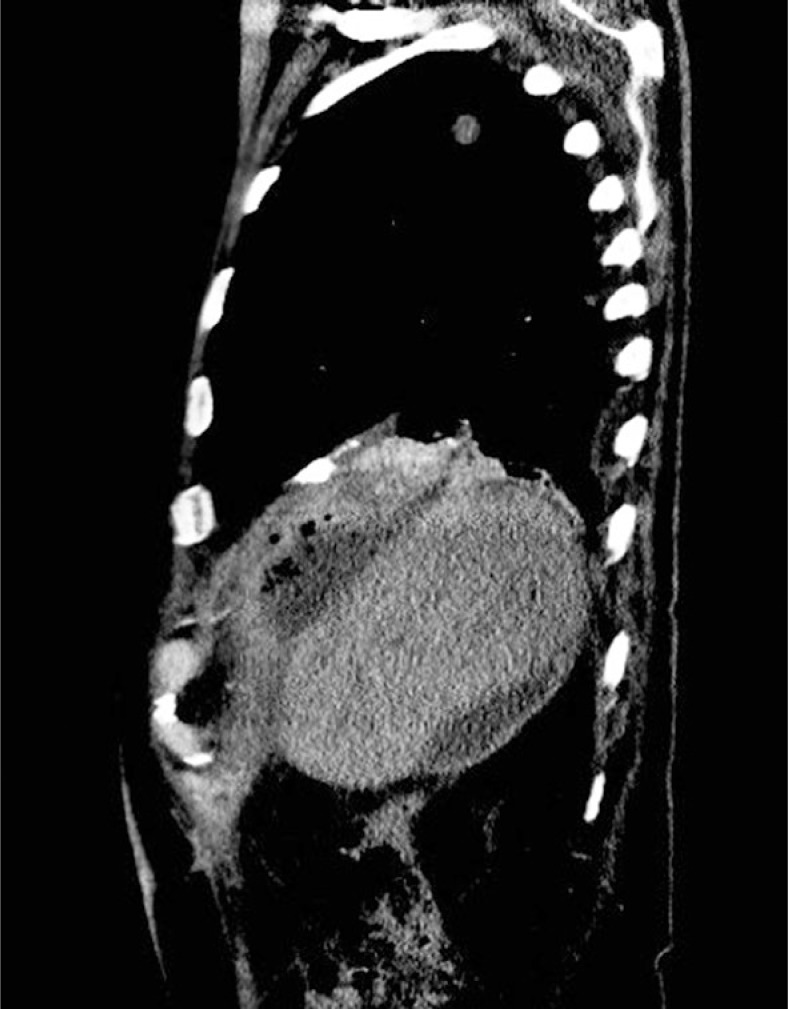
Bronchobiliary fistula (BBF) demonstrated by contrast enhanced CT.

In cases when a clinical suspicion for BBF or PBF exists, the first dilemma is which diagnostic method should be used to either confirm or refute our suspicion. The possible diagnostic methods listed in the literature are bronchoscopy^2^,^21^, bronchogram^2^,^4^, CT^3^,^21^ (Figure 1) and MRI^21^ that are routinely used in every clinical praxis^26^,^27^, cholescintigraphy^4^,^28^, magnetic resonance cholangiopancreatography (MRCP)^3^,^28^, percutaneous transhepatic cholangiography (PTC)^2^,^3^, endoscopic retrograde cholangiopancreatography (ERCP)^2^,^3^,^28^, and fistulography in the case of a biliocutaneus fistule.^2^,^4^ Most authors agree that bronchoscopy and bronchogram are not sensitive enough. From the remaining methods, PTC, ERCP and fistulography of the biliocutaneus fistula (if present) are stated as the most sensitive by most of the articles reviewed.^2^–^4^,^28^ The latter being described as the method of choice for TBF confirmation by some articles.^2^,^4^ The advantage of ERCP is the additional therapeutic possibility in the case of the biliary tract obstruction. Cholescintigraphy (hepatobiliary iminodiacetic acid – HIDA, paraisopropyl iminodiacetic acid – PIPIDA or diisopropyl iminodiacetic acid – DISIDA scan), CT, and MRI are less sensitive methods. Intraoperatively, a fistula can be demonstrated with the intraoperative cholangiography.

Regarding the therapy there are three possible approaches to treat TBF: surgical, conservative, and the combined approach. The bulk of the literature advocates the surgical approa ch.^1^,^2^,^4^,^5^,^7^,^10^–^14^,^17^,^18^,^20^,^21^,^23^,^24^,^29^ In 1967, Ferguson and Burford^1^ published an article on TBF, summarizing the basic steps necessary for a successful TBF treatment:

early aggressive treatment by thoracotomy,
adequate subcostal drainage of the hepatic bed under direct vision,
secure closure of the diaphragmatic perforation by non-absorbable sutures,
decortication for pleurobilia, if necessary lobectomy for bronchobiliary fistula and
the awareness of the need for prophylactic decompression of the biliary tree.

In the next decades other authors have published their experience in treating TBF. The studies reviewed included from 2 to 123 cases. 1,2,4,5,7,10–14,16,18–25 By comparing these studies, one can notice that non-traumatic TBF requires more operations for the successful treatment than traumatic TBF. This statement is best illustrated by the publications of Warren *et al*.^2^ and Gugenheim *et al*.^4^ In the first case 15 patients have been cumulatively operated 63 times and in the second case 16 patients have been operated 42 times. In contrast, Oprah and Mandal^12^ report of only 17 operative interventions needed for the successful treatment of 14 patients with traumatic TBF. The common denominator in TBF treatment is to remove or solve the abdominal pathology that caused TBF formation. Additionally, adequate drainage of the subphrenic space and a secure closure of the diaphragmatic defect are of importance. Regarding the need for decortication or lobectomy, the authors do not share the same opinion. A lot of them think that lobectomy is necessary only occasionally, if the lung tissue is chronically inflamed or damaged^2^,^4^,^7^ to the point that it could cause problems in the future.

According to the literature the possible ways of TBF closure are: non-absorbable sutures^1^, the use of a mesh^30^,^31^, AlloDerm®^3^, pericardial fat tissue flap^17^, pleural flap^5^, and the use of a vascularized pedicel of intercostal muscle.^17^

In the last decade of the 20^th^ century reports of conservative ways of TBF management started to appear. In these cases the treatment was comprised of biliary drainage using PTC (percutaneous transhepatic biliary drainage – PTBD) or ERCP and percutaneous drainage of the subphrenic, subhepatic or intrahepatic abscess if it existed. When performing ERCP there is the additional option of endoscopic sphincterotomy (EST) and stent placement or nasobiliary drainage (NBD).^32^ The goal of these interventions is to minimize the pressure in the biliary tree, drain an abscess if present, prevent the flow of bile through the TBF and enable the healing of the TBF. In 1996 Yilmaz *et al*.^15^ published a series of 11 cases of BBF after hydatid cyst operation. In all 11 patients an ERCP with nasobiliary drainage was performed, which lead to fistula closure. In some articles histoacyrl embolization of the TBF is stated as an additional option to the procedures listed above. Richter *et al*.^33^ report of successful histoacyrl occlusion of a BBF after a selective duct cannulation during ERCP. Kim *et al*.^34^ report of a successful histoacyrl embolization of BBF under bronchoscopic guidance. According to some authors, there is also the option of applying octreotide – a somatostatin analogue which is thought to reduce the secretion through enteral and biliary fistulas and thus promote their healing.^19^,^22^,^35^ But it should be kept in mind that there were reports of an increased number of septic and thrombotic complications while using octreotide.^36^

The combined approach uses biliary drainage (via ERCP or PTC) and abscess drainage (US or CT guided) in the first step, followed by a delayed surgical intervention. The reason for such a course of action are patients who are initially not candidates for surgery (ARDS, sepsis, comorbidity, etc…)^3^ and patients where non-surgical interventions have failed.^8^,^17^,^19^,^22^,^28^

## Case report

The patient was referred to our medical centre in September 2009. At the time he was 73 years old. In the past an elective cholecystectomy for cholecystolithiasis was performed. He had essential arterial hypertension (regulated), suffered from a myocardial infarction, and in his youth he also had pulmonary TBC. In July 2009 a right hemicolectomy for colon carcinoma (stage T3N0M0) was performed at a regional general hospital. Preoperatively, pleural thickening in the left apical and right basal part of the lung was described on chest x-ray (due to TBC during his youth).

On follow-up examinations, the progression of the disease (using US and CT) with two metastases in the right hemiliver was diagnosed. One was located in the deep of the right anterior section (border of 5^th^ and 8^th^ segment) and was 4 cm in size. The other was located in the 7^th^ segment and was 2 cm in size. There were also two metastases found in the lungs; one in the 2^nd^ left and the other in the 6^th^ right segment. At the multidisciplinary team meeting it was decided that liver surgery should be attempted first. A formal anatomical right hemihepatectomy was planned. In the beginning of October 2009 the patient was operated. With intraoperative inspection and US we found the two tumours in the right hemiliver as depicted by CT. Unexpectedly we also found two metastases in the left hemiliver, located in the 3^th^ and 4^th^ segment. Both were 1 cm in diameter and were not visible on the preoperative CT scan. A metastasectomy in the 3^rd^ and 4^th^ liver segment was performed. The remaining two metastases in the 7^th^ segment and right anterior liver section were treated with radiofrequency ablation (RFA). A few days after the surgery the patient reported malaise and increasing pain in the right upper part of the abdomen. His body temperature and inflammatory parameters were elevated. A control US showed a hypoechogenic fluid collection in the area of the liver where RFA had been performed. ERCP demonstrated a biliary leak from the right anterior section into the cavity after RFA, endoscopic papillotomy (EPT) was performed and US-guided puncture and drainage was done, which revealed the fluid to be an infected biloma. Despite drainage and the broad spectrum antimicrobial therapy, the patient’s state deteriorated. Pneumonia of the right lower and middle lung lobes and a biliocutaneus fistula developed. A reoperation was performed, which included the evacuation and lavage of the abscess cavity, sutures at the area of biliary leakage and the placement of two drains. After the intervention the patient’s state promptly improved. At the end of November 2009 he was discharged from our hospital, with the intention of the further oncological and surgical therapy.

In January 2010 the patient was again urgently admitted to our department. He was complaining of pain in the upper right part of the abdomen and pleuritic pain in the lower right part of the thorax. His body temperature was 39°C and he had a productive cough with yellowish sputum. White cell blood count and CRP were elevated and shadowing was identified in the lower right part of the lungs on chest x-ray. A CT scan of the thorax and abdomen was performed, showing a subphrenic abscess with a BBF. Again an operative evacuation, lavage and drainage of the abscess were performed. Sutures were placed at the site of biliary leakage and at the abdominal ostium of the BBF. A right hepatectomy was not performed, because the remaining liver volume would have been too small. Instead, a right portal vein embolization (PVE) was planned. We speculated that this would lead to atrophy of the right hemiliver, cessation or limitation of biliary leakage and compensatory left liver hypertrophy. The patient was discharged with a drain. The right PVE mentioned followed and his case was presented at an oncological council. Because the number of pulmonary metastases increased, the conclusion of the council was that he should receive palliative oncological therapy.

**FIGURE 2.**
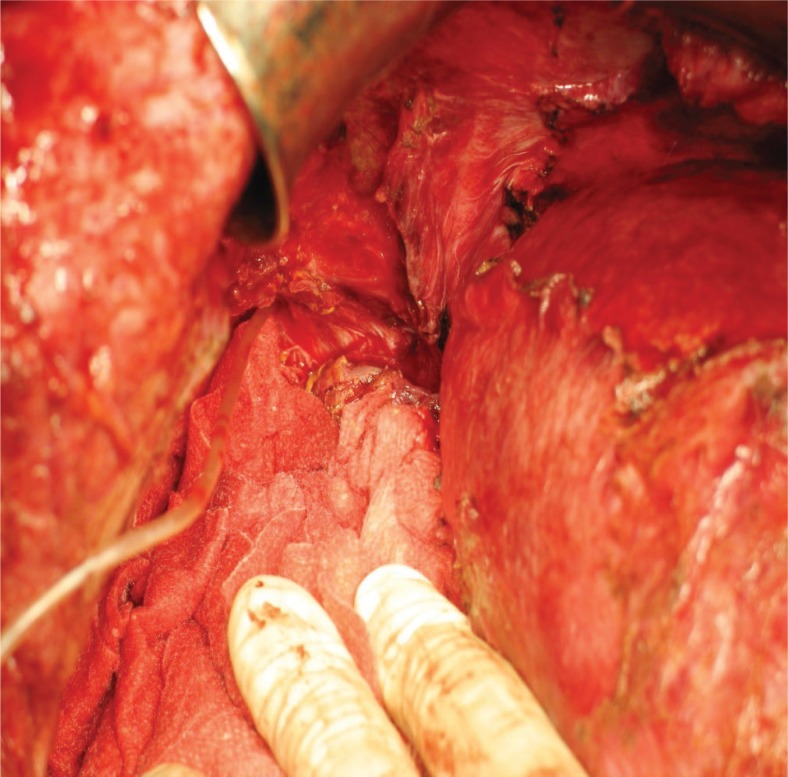
Placing of the catheter through the defect diaphragm.

**FIGURE 3.**
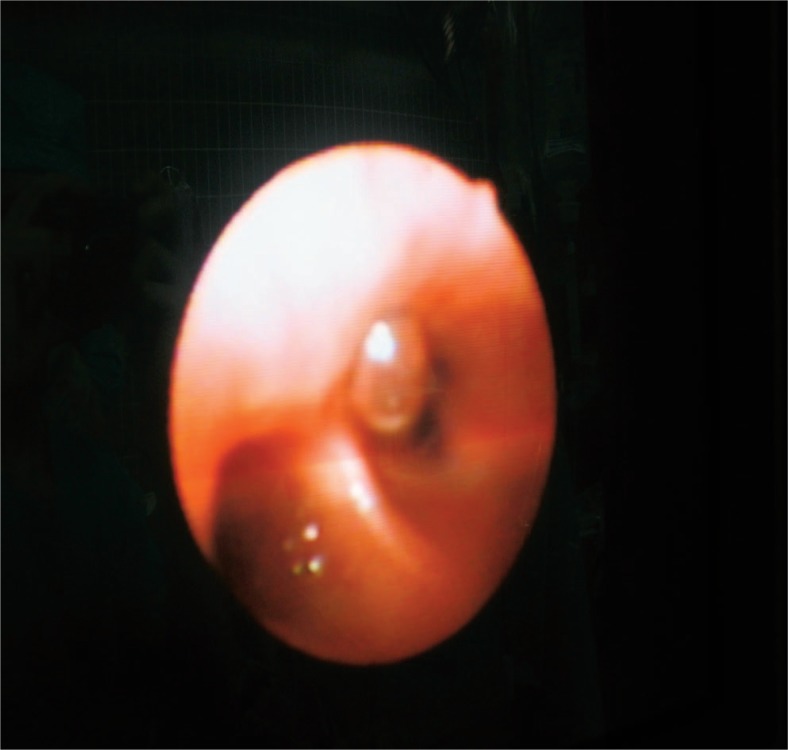
Visualizing the tip of the catheter with a bronchoscope in the 8th segment of the right lung.

**FIGURE 4.**
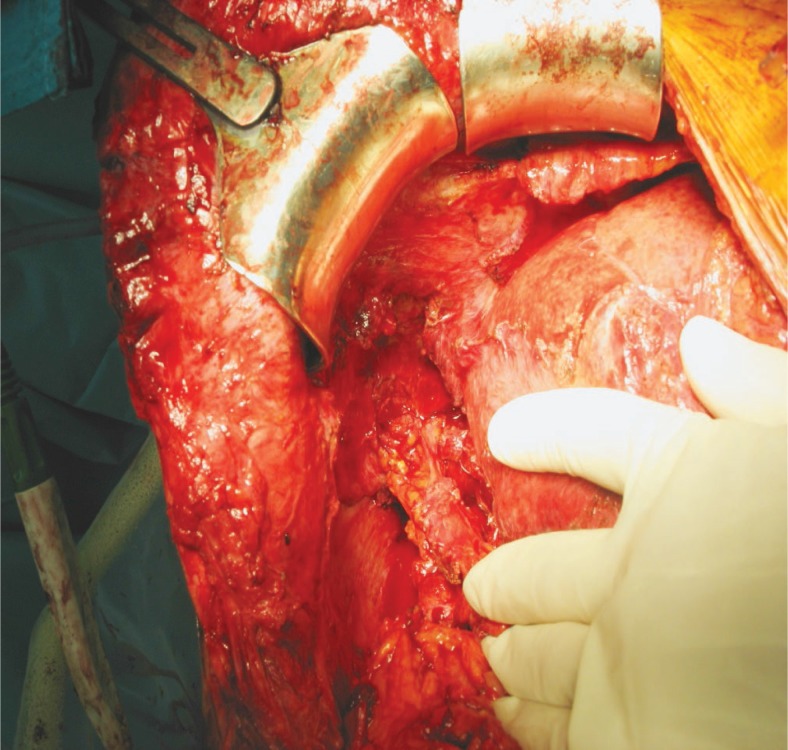
Placing of the omentum majus into the ostium of the BBF and closing the defect.

In August 2010 he was again admitted with malaise, intermittent fever, and cough with yellowish sputum. On CT scan the abscess in the remaining right liver was again present. As it was speculated, the right hemiliver was atrophic and the left liver was hypertrophic as a result of right PVE. The BBF seen on the previous CT scan was still present. Despite the pulmonary progression, we decided to perform a right hepatectomy with the intention to eliminate the inflammatory focus which was the reason for the BBF. Decisive for this step was the left liver hypertrophy taking place after the right PVE. After the right hepatectomy, we intraoperatively confirmed the presence of the BBF by placing a small diameter catheter through the abdominal ostium (Figure 2) and visualizing the tip of the catheter with a bronchoscope (Figure 3). Because of the pulmonary metastatic progression we did not decide to excise the fistula and the right lower lung lobe. Instead we closed the abdominal ostium of the BBF with a part of the omentum majus (Figure 4).The omentum was sutured over the abdominal opening of the BBF and was also used to cover the resection surface of the remaining left hemiliver, but was not sutured to it. After lavage a drain was placed. Postoperatively, a prompt decrease of the white cell blood count and CRP, cessation of bilioptysis and the improvement of the patient’s general status was recorded. On follow-up examinations there was no sign of BBF recurrence. The patient expired in March 2011 due to the pulmonary metastatic progression of the disease.

## Discussion

By comparing our case to the literature reviewed, one can notice several parallels. Our case describes an iatrogenic BBF. In fact more cases of TBF in the developed world are probably due to trauma and iatrogenic causes than due to liver infection (echinococcic amoebic or pyogenic). Pathogenesis and the formation point of BBF described, coincides with the reviewed papers. The clinical suspicion was in our case confirmed with contrast enhanced CT of the thorax and upper abdomen. According to the literature this is not the most sensitive method. ERCP offers better results and has the additional advantage of biliary tree decompression as shown above. Intraoperative, TBF can be confirmed with cholangoigraphy. In the case of the liver resection, as it was shown in our clinical case, it can also be confirmed by visualizing a small diameter catheter put through the abdominal ostium with a bronchoscope.

The biggest dilemma regarding TBF is the proper treatment. Although the bulk of the literature advocates surgery, non-surgical or conservative therapy is a good alternative in some cases. As shown by Yilmaz *et al*.^15^, Singh *et al*.^19^ and Ertugrul *et al*.^37^, the conservative therapy with biliary and abscess drainage can be effective in cases where TBF is the result of liver abscess, complicated liver hydatidosis after surgical cyst removal, biliary tract obstruction due to stones or short strictures and in selected cases of posttraumatic TBF.

For the rest, surgery is probably the best solution. The basic steps outlined by Ferguson and Burford^1^ are a good basis but need some comment. Thoracotomy is necessary if the preoperative investigation indicates the need for the lung resection^4^,^14^ or in the case of posttraumatic TBF.^4^ Laparotomy is mandatory if biliary tract obstruction which cannot be managed conservative is the cause of TBF.^2^,^4^ In cases of extensive disease two separate incisions (approaches) are probably superior to thoracoph-renolaparotomy.^14^ Regarding the diphragmal defect closure, we can add our report of BBF closure with omentum majus to those listed above. In our opinion preoperative biliary decompression would act beneficial on TBF therapy outcome in all TBF cases not just those caused by the biliary tract obstruction.

In our case two operations were needed for a successful BBF treatment. Despite the palliative nature of the treatment a surgical approach was chosen. The reason was the persistence of biliopytisis and recurrent septic bursts, although the conservative treatment with ERCP, EPT and US guided abscess drainage had been done first. We think that biliary leakage into the cavity produced by RFA was the reason for failure of the conservative measures. The only possible solution was the right hepatectomy, which removed the inflammatory focus and closure of the fistula as described above. Though this might seem an extensive treatment for a patient with a poor prognosis, it was the only way of improving his quality of life.

## Conclusions

Thoracobiliary fistulas are pathological communications between the biliary tract and the bronchial tree or the biliary tract and the pleural space. The first are termed bronchobiliary and the second pleurobiliary fistula. Etiologically, they can be divided into congenital TBF, TBF resulting of liver hydatid disease or liver abscess, biliary tract obstruction, traumatic TBF, and iatrogenic TBF. The article summarizes the characteristics of the clinical picture and laboratory findings of BBF and PBF. The most sensitive methods for TBF confirmation are ERCP, PTC, and fistulography of a biliocutaneus fistula, if it exists. Opinions regarding the therapy differ. Although the bulk of the literature advocates surgical therapy, newer papers report of successful non-surgical therapy. These interventions are comprised of a biliary drainage via ERCP or PTBD, percutaneous US or CT guided drainage of a subdiaphragmatic, subhepatic or intrahepatic abscess if it exists and ERCP or bronchoscopic guided fistula embolization. The authors presented the fistula closure with the greater omentum as a possible method of TBF closure which was according to the authors’ knowledge not reported on previously.

## Figures and Tables

**TABLE 1.** Case and retrospective studies of thoracobiliary fistulas (TBF)

**Authors**	**Year**	**No. of patients**	**Aetiology**	**Fistula type**		**Initial therapy**	**Second line therapy**	**Outcome**	**Special recommendations**
Ferguson and Burford^1^	1967	7	Trauma (blunt) 4x Abscess (pyogenic) 2x Biliary obstruction 1x	PBF BBF BBF		**Surgical**		No recurrence of TBF	The paper summarizes the basic steps for a successful surgical treatment of TBF
Saylam *et al*.^10^	1974	6	Echinococcosis 2x Abscess (pyogenic) 2x Not known 2x	TBF		**Surgical** : Thoracic approach, 2 patient refused surgery		1 patient died of septic shock	Advocates thoracotomy. Biliary obstruction if present, should be resolved first
Boyd^7^	1977	16	Iatrogenic bile duct stricture	PBF and BBF		**Surgical** : Stricture correction, only 1 lobectomy		Not clearly stated	Stricture correction and subdiaphragmatic drainage are needed for TBF healing
Tierris *et al*.^11^	1997	3	Echinococcosis	BBF		**Surgical**	**Repeated surgery** in 1 patient	1 patient died due to massive PE	A case of left liver and left lung BBF in described.
Oparah and Mandal^12^	1978	4	Trauma (penetrant)	PBF		**Surgical** : Thoracotomy, +/− laparotomy. In 2 instances only a chest tube was inserted.		No recurrence. Patients with only tube thoracostomy had a prolonged hospital stay	Tube thoracostomy without surgery is indicated only if instituted early in combination with adequate subphrenic drainage
Wei *et al*.^13^	1982	2	Bile duct obstruction (stones)	BBF		**Surgical** : Abdominal approach		No recurrence of BBF	
Warren *et al*.^2^	1983	15	Bile duct obstruction: Iatrogenic stricture 10x Congenital 1x Trauma 1x Stones 2x	BBF 13x PBF 2x		**Surgical** : Abdominal approach. Only 1 lobectomy	**Repeated surgery** in 9 patients	63 operations in total. Eventually all recovered.	Advocates abdominal approach for TBF that are the result of biliary tract disease
Caporale *et al*.^14^	1987	30	HDL	TBF Uses a different classification		**Surgical** : Thoracotomy, laparotomy or thoracophrenolaparotomy, with lung resection, cyst and pericyst removal, TBF resection, suturing of the diaphragm and subphrenic drainage for 2 to 4 weeks.	**Repeated surgery** in 2 patients	3 patients died − 10.3% (2 of haemorrhagic hock, one PE) 2 patients had a recurrence of TBF	Thoracotomyifpreoper ativestudiesshowirreve rsiblelungimpairmenta ndthelivercyst is single. Laparotomy if the abdominal disease is prevalent. Forextensivediseasep erformtwoseparatein cisionsratherthenperf ormingthoracophrenolaparotomy.
Gugenheim *et al*.^4^	1988	16	Iatrogenic bile duct obstruction 8x Echinococcosis 7x Abscess (amoebic) 1x	BBF		**Surgical** : Abdominal approach	**Repeated surgery**	42 operations total Eventually all recovered	Abdominal approach for TBF that result from biliary tract disease. Thoracic approach for traumatic TBF and if lung resection is planned
Yilmaz *et al*.^15^	1996	11	Complicated liver hydatidosis (previously operated) 8x Iatrogenic bile duct stricture 1x HDL + bile stones 1x Abscess (amoebic) 1x	BBF		**Conservative**: NBD in 4 patients EST + NBD in 7 patients	**Repeated Conservative treatment** in 3 cases. Prolonged NBD + stent insertion in 1 case	All recovered	First successful case series of nonsurgical treated BBF
Senturk *et al*.^16^	1998	3	Alveolarhydatid disease (AHD) 1x AHD (after surgical cyst removal) 1x AHD (after surgical cyst removal)+ TBC 1x		BBF	**Conservative**: ERCP +EST after unsuccessful surgery (1st case) ERCP +NBD (2nd case) Anti-tuberculotics only (3rd case)	**Repeated Conservative treatment**: Octreotid (1st case) Stent (2nd case)	Recurrence in all cases. At the time of publication one patient was bed-ridden	Treatment of BBF due to AHD is unsatisfactory by either surgery or nonsurgical therapy. The reason is probably the more invasive nature of AHD
Chua *et al*.^17^	2000	2	Iatrogenic		BBF	**Conservative**: Abscess and biliary drainage (1st case) **Surgical** with thoracotomy, fistula and lung resection (2nd case)	**Surgical**: after BBF recurrence in 1st case	Both patients recovered	Describes the use of a vascularized intercostal muscle pedicle and a pericardial fat pad as a way of fistula closure.
Kabiri *et al*.^18^	2001	123 (cases of thoracic rupture of HDL)	HDL		BBF – confirmed in 50 cases by biliopytisis	**Surgical**: Postero-lateral thoracotomy with trans diaphragmatic cyst removal.		11 patients died (8.9%), only 1 recurrence	Advocatestransthoraci csurgerywithpreoperati veendoscopicsphincte rotomy
Singh *et al*.^19^	2002	8	Abscess : Amoebic 3x Pyogenic 1x Trauma 3x Iatrogenic 1x (after PTC)		BBF BBF BBF(1x), PBF (2x) PBF	**Conservative** (7 cases) EST+ Abscess or pleural drainage + octreotide **Surgical**(1 case) Iatrogenic biliary stricture repair	**Surgical** (2 cases)	All recovered	TBF may be successfully managed conservative. Surgery reserved for failure of this approach. Routinely uses octreotide
Gerazounis *et al*.^20^	2002	21	Echinococcosis		BBF	**Surgical**: Right posterolateral thoracotomy.		2 patients died − 9.5%. All remaining patients recovered	Advocates surgery in cases of complicated echinococcosis with BBF
Uchikov *et al*.^21^	2003	3	HDL 2x Abscess (echinoccocic) 1x		BBF	**Surgical**: Right thoracophrenotomy		All recovered	
Ong *et al*.^22^	2004	2	Bile duct obstruction Stones 1x Iatrogenic 1x		BBF	**Conservative**: ERCP +EPT + stent+ octreotide	**Surgical**: in 2nd patient	1 death due to v. cava inf. laceration	Advocates the use of octreotide
Peker *et al*.^23^	2007	4	HDL		BBF	**Surgical**(2cases) **Conservative** (2 cases)		All recovered	Proposes a BBF treatment algorithm
Tocchi *et al*.^24^	2007	31	HDL		BBF (23 histologically cofirmed)	**Surgical** Lung resection was required in 25 cases		3 patients died (9.6%) 26 patients recovered	Advocates thoracoabdominal incision (approach)
Eryigit *et al*.^5^	2007	3	Abscess (echinoccocic) 2x Trauma (penetrant) 1x		BBF	**Surgical** with thoracotomy and lung resection in two cases		No recurrence reported	
Aydin *et al*.^25^	2009	3	Abscess (echinoccocic) 1x Iatrogenic 2x		BBF	**Conservative**: Percutaneous drainage with EPT + stent placement or NBD.		No recurrence reported	Advocates conservative approach. Embolization of the fistula is described.

BBF = bronchobiliary fistulas; PBF = pleurobiliary fistulas; HDL = hydatid disease of the liver; ERCP = endoscopic retrograde cholangiopancreatography; EPT = endoscopic papilotomy; NBD = nasobiliary drainage; PE = pulmonary embolism
